# An expanded parenchymal CD8+ T cell clone in GABA_A_ receptor encephalitis

**DOI:** 10.1002/acn3.50974

**Published:** 2020-01-14

**Authors:** Aline Bracher, Carmen Alcalá, Jaime Ferrer, Nico Melzer, Reinhard Hohlfeld, Bonaventura Casanova, Eduardo Beltrán, Klaus Dornmair

**Affiliations:** ^1^ Institute of Clinical Neuroimmunology Biomedical Center and Hospital of the Ludwig‐Maximilians Universität München Munich Germany; ^2^ Department of Neurology Hospital Universitari i Politècnic la Fe Valencia Spain; ^3^ Department of Pathology Hospital Universitari i Politècnic la Fe Valencia Spain; ^4^ Clinic of Neurology and Institute of Translational Neurology University Hospital Münster Münster Germany; ^5^ Munich Cluster for Systems Neurology (SyNergy) Munich Germany

## Abstract

The role of T cells in autoimmune encephalitis syndromes with autoantibodies against cell surface antigens is still enigmatic. Here we analyzed the T cell receptor repertoires of CD8+ and CD4+ T cells in a patient with “idiopathic” gamma‐aminobutyric‐acid‐A receptor (GABA_A_‐R) encephalitis by next‐generation sequencing and single‐cell analyses. We identified a CD8+ T cell clone that was strongly expanded in the cerebrospinal fluid and in the hippocampus but not in the operculo‐insular cortex. By contrast, CD4+ T cells were polyclonal in these tissues. Such a strong clonal expansion suggests that CD8+ T cells may play a significant role in the pathogenesis.

## Introduction

Autoimmune encephalitis syndromes are rare and severe diseases with acute inflammation of the central nervous system.^1^, ^2^, ^3^, ^4^ A shared feature in all forms of autoimmune encephalitis is the presence of autoantibodies, but two groups may be distinguished as to the target antigens of the autoantibodies. In one group, intracellular antigens, such as Hu, Ri, Ma/Ta, or GAD65 are recognized.^2^ Here it is assumed that T cells may play a major pathogenic role in development of autoimmunity because intracellular antigens are initially not accessible to antibodies but may be released from cells killed by T cells. The release of cryptic (tumor) epitopes may then initiate B cell responses and autoantibody production. In the other group, the autoantibodies recognize extracellular domains of membrane proteins on cells of the central nervous system. This recognition exerts direct effects on the function and cellular localization of these antigens rendering these autoantibodies directly pathogenic. In this group of autoimmune encephalitis, the role of T cells in the pathogenesis is still unclear.

Recently, encephalitis associated with autoantibodies against the gamma‐aminobutyric‐acid‐A receptor (GABA_A_‐R) has been described.^5^, ^6^, ^7^ These autoantibodies bind to GABA_A_‐R and cause selective reduction of postsynaptic GABA_A_‐R clusters at inhibitory synapses, hyperexcitability of inhibitory neurons, and severe neurological symptoms.^6^, ^7^, ^8^, ^9^

Here we investigated the intrathecal and parenchymal T cell receptor (TCR) repertoire in a paradigmatic case with “idiopathic” GABA_A_‐R encephalitis where we had access to cerebrospinal fluid (CSF) and freshly frozen autopsy samples. Therefore we could analyze the TCR repertoire in depth by next generation sequencing (NGS) and isolate single cells by laser capture microdissection (LCM) for analyzing their matching TCR *α*‐ and *β*‐chains. In this well characterized “index patient 2” (IP2), where no reactivity against viral antigens has been detected but a high titer of autoantibodies against GABA_A_‐R,^6^ we identified a CD8+ T cell clone (termed TCR‐IP2) that was strongly expanded in the CSF and in the hippocampus but not in the operculo‐insular cortex (OIC), which was included as control. Of note, we could not detect any expanded CD4+ T cell clones. Although it is beyond doubt that autoreactive antibodies are major drivers of disease pathogenesis in this form of autoimmune encephalitis, our results suggest that CD8+ T cell clones may contribute to the pathogenesis of GABA_A_‐R encephalitis.

## Materials and Methods

### Patient IP2

A detailed case history of the 51‐year‐old male patient IP2 is described.^6^ No evidence of precipitating viral encephalitis was found. The patient died after medication with several anticonvulsants and immunosuppressants. The CSF sample was collected briefly before death. Peripheral blood cells were not preserved. Samples were acquired after informed consent. The study was approved by the ethics committee of the University of Valencia.

### T cell receptor repertoire analysis of autopsy tissue by NGS

The TCR repertoire from autopsy tissue (hippocampus and OIC; five consecutive frozen sections of 10 *µ*m from each, diameter ~ 8mm) was analyzed as described.^10^ RNA was extracted using the PureLink RNA kit with on‐column digestion of DNA by PureLink DNase I according to the manufacturer's instructions (ThermoFisher, Darmstadt, Germany). Illumina MiSeq sequencing was performed at FISABIO, Valencia, Spain.

### Isolation of single T cells, and TCR analysis from brain parenchyma and CSF cells

For isolation of single cells from brain tissue, LCM was performed as described.^11^ See Data S1 for details.

To isolate single cells from CSF, we separated CD4+ and CD8+ T cells from CSF by magnetic beads (Miltenyi, Bergisch‐Gladbach, Germany) and isolated single cells manually. TCR *α*‐ and *β*‐chains isolated either by LCM or manually were analyzed by multiplex PCR using an unbiased set of primers^12^, ^13^ or clone‐specific primers (Table S1).

### Immunohistochemistry

For IHC, we used FFPE fixed brain tissue sections for triple staining for CD3, 4′,6‐diamidin‐2‐phenylindole (DAPI), and either CD4 or CD8. For triple staining for CD8, perforin, and DAPI, we used frozen tissue sections. See Data S1 for details.

## Results

### Repertoire analyses reveal an expanded CD8+ T cell clone in the CSF

**Figure 1:**
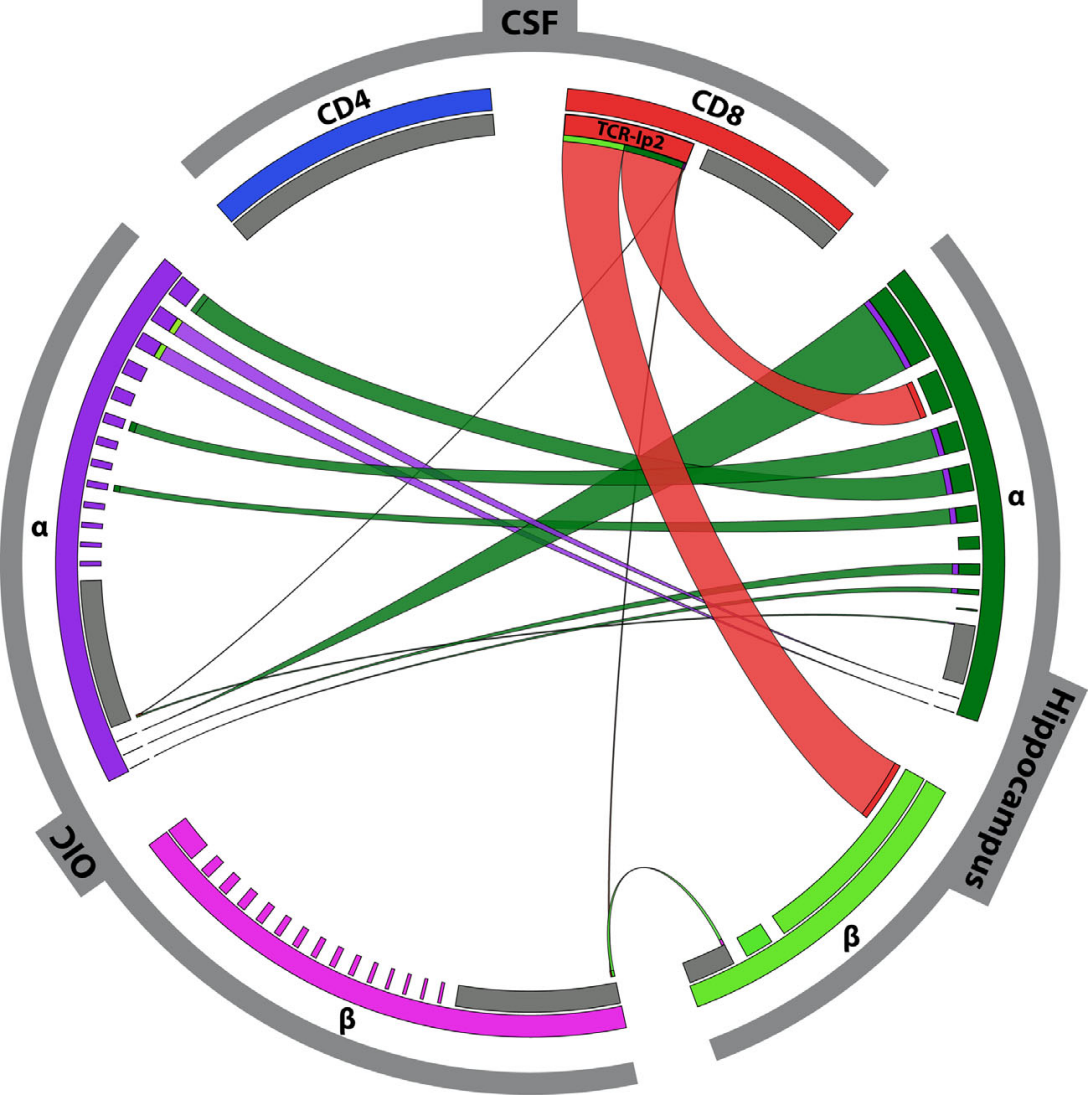
TCR repertoire analysis in CSF and brain tissue. Repertoires of TCR *α*‐ and *β*‐chains from CSF cells are at the top of the circle. TCR repertoires are shown in blue for CD4+ cells (upper left) and in red for CD8+ cells (upper right). For the TCR repertoire of OIC (left) and hippocampus (right), individual repertoires for *α*‐chains (upper half) and *β*‐chains (lower half) are shown. In the OIC, the *α*‐chains are shown in purple, and the *β*‐chains in pink. In the hippocampus, the *α*‐chains are shown in dark green and the *β*‐chains in light green. CD4 and CD8 repertoires could not be distinguished in the tissue homogenates. The widths of the colored segments in the inner circle indicate the relative abundance of each T cell clone. The polyclonal background in each sample is depicted in grey. Expanded T‐cell clones shared between the compartments are visualized as semi‐circular connections. The segment including the *α*‐ and *β*‐chains AV13‐1‐CAAS‐WG‐TGNQFYF‐AJ49 and BV12‐3 CASS‐AGG‐‐DTQYF‐BJ2‐3 of clone TCR‐IP2 is labeled in red. Repertoires were generated by combining data from NGS and single cell analysis. Images were generated using the CIRCOS software (http://circos.ca/).

To identify disease‐related T lymphocytes, we investigated the TCR repertoire from CSF of patient IP2 by single cell analyses. The low numbers of cells impeded NGS analysis of CSF cells. Therefore, we separated CD4+ and CD8+ T cells from CSF by magnetic beads and analyzed the TCR repertoires by multiplex PCR and Sanger sequencing (Fig. 1, top). The *α*‐ and *β*‐chain repertoires of CD3+CD4+ cells from CSF were polyclonal with no hints for any clonal expansions. From 40 single cells, we identified 11 matching *αβ*‐pairs and 4 *β*‐chains, which were all different (Table S2). In contrast, in the CD3+CD8+ population from CSF, we detected strong expansions of distinct *α*‐ and *β*‐chains. From 48 single T cells we identified 11 matching TCR *αβ*‐pairs and 2 *β*‐chains (Table S3). Strikingly, in 6 out of the 11 cells for which we could detect matching *α*‐ and *β*‐chains, we found identical AV13‐1‐ and BV12‐3‐chains. This suggests a strong expansion of a CD8+ T cell clone, which we termed TCR‐IP2.

### The expanded T cell clone is also present in the hippocampus

To examine whether T cell clone TCR‐IP2 was also present in brain tissue, we analyzed tissue homogenates from hippocampus and OIC by NGS. After bioinformatic analysis, the total number of unique detected reads with functional CDR3 was 1740 TCR *α*‐ and 537 *β*‐chains in the hippocampus and 2396 TCR *α*‐ and 1053 *β*‐chains in the OIC.

In parallel, we double‐stained tissue sections from hippocampus and OIC for CD3 and CD4 or CD8, isolated double positive cells by LCM, and analyzed their TCR chains. From 177 single CD3+CD8+ T cells isolated from the hippocampus we found the *α*‐chain of TCR‐IP2 in 19, and the *β*‐chain in 22 cells (Fig. 1, below, right). We could not detect the *α*‐ or the *β*‐chain of TCR‐IP2 in any of 111 CD3+CD8+ T cells isolated from OIC, and we identified only one read of each chain by NGS (Fig. 1, below, left). Thus, AV13‐1‐ and BV12‐3‐chain expansions were pronounced in the hippocampus, but only marginal in the OIC. These data provide evidence that T cell clone TCR‐IP2 was specifically expanded in the hippocampus and CSF but not in the OIC.

**Figure 2:**
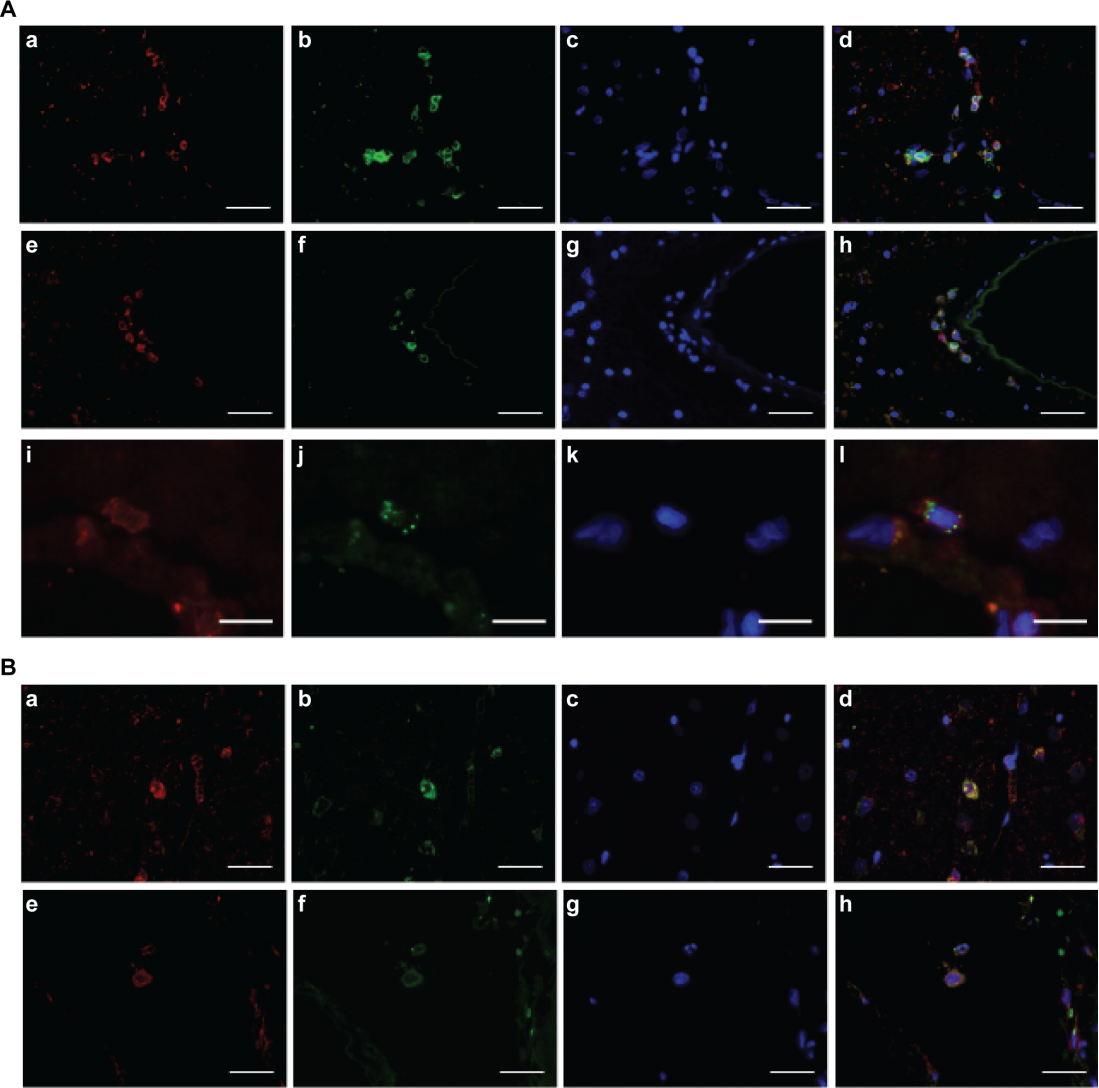
Immunohistochemistry reveals infiltration of CD8+ and CD4+ T cells in brain tissue of patient IP2. (A) CD8+ T cells infiltrating the hippocampus and OIC. a to h: FFPE tissue sections were stained for CD3 (red), CD8 (green), DAPI (blue), and merged. Row #1 (a to d) shows staining of a parenchymal region in the hippocampus. Row #2 (e to h) shows staining of a perivascular region in the OIC. A blood vessel is visible in the right half of the figure. The left and middle panels show single color staining for each of the antigens CD3 (a, e), CD8 (b, f), and DAPI (c, g). The right panels show a three‐color overlay of CD3, CD8, and DAPI (d, h). Scale bars: 25 *µ*m. Row 3 (i to l): CD8+ T cells in a parenchymal region of the hippocampus express the activation marker perforin. Frozen tissue sections were stained for CD8 (i, red), perforin (j, green), DAPI (k, blue), and merged (l). Scale bar: 10 *µ*m. (B) CD4+ T cells infiltrating the hippocampus and OIC. a to h: FFPE tissue sections were stained for CD3 (red), CD4 (green), DAPI (blue), and merged. Row #1 (a to d) shows staining of a parenchymal region in the hippocampus. Row #2 (e to h) shows staining of a perivascular region in the OIC. The left and middle panels show single color staining for each of the antigens CD3 (a, e), CD4 (b, f), and DAPI (c, g). The right panels show a three‐color overlay of CD3, CD4, and DAPI (d, h). Scale bars: 25 *µ*m.

Analysis by immunohistochemistry revealed that CD8+ cells outnumbered CD4+ T cells in the hippocampus as well as in OIC (Table 1). CD3+CD8+ infiltrates were located predominantly in the parenchyma of the hippocampus (Fig. 2A, a–d), and in perivascular regions of the OIC (Fig. 2A, e–h). We detected expression of the activation marker perforin in about 50 percent of the parenchymal CD8+ T cells in the hippocampus. An exemplary double‐staining for CD8 and perforin is shown (Fig. 2A, i–l). The rare CD3+CD4+ cells were preferentially detected in the perivascular space in the hippocampus and OIC (Fig. 2B, a–h). Thus, immunohistochemistry supports the repertoire analyses in suggesting an encephalitogenic role of activated CD8+ T cells, in particular of clone TCR‐IP2, in the hippocampus of patient IP2.

**Table 1:** Cell densities per square cm of CD3^+^, CD4^+^, and CD8^+^ cells in the hippocampus and operculo‐insular cortex of patient IP2 as determined by immunohistochemistry.

	CD3^+^ cells/cm^2^	CD4^+^ cells/cm^2^	CD8^+^ cells/cm^2^
Hippocampus	77	10 (13%)	67 (87%)
Operculo‐insular cortex	147	29 (20%)	118 (80%)

## Discussion

Analysis of the TCR repertoires in the CSF, the hippocampus, and the OIC of patient IP2 revealed a virtually monoclonal expansion of the CD8+ T cell clone TCR‐IP2 in the CSF and the hippocampus but not in the OIC. By contrast, CD4+ T cells were polyclonal in the CSF and in the hippocampus and infrequent in the OIC. Expansion of a single CD8+ T cell clone in CSF and hippocampus is surprising because lymphocyte expansions in neuroinflammatory disorders are mostly oligoclonal, that is, they are composed of several distinct clones. We can exclude that the observed expansions are due to biased PCR efficiencies as the repertoire of CD4+ T cells, studied with identical methods was found to be polyclonal. Oligoclonal expansions of T cells are commonly observed in Rasmussen encephalitis,^14^, ^15^ MS,^11^, ^16^ and paraneoplastic encephalomyelitis,^17^, ^18^ whereas—to our knowledge—monoclonal expansions have not yet been described in neurological autoimmune diseases except a single case of autoimmune myositis mediated by a *γδ* T cell clone.^19^

So far, our study is limited to a single index patient because GABA_A_‐R encephalitis is an extremely rare disease and samples from brain tissue are generally not available. Moreover, we applied techniques that require preservation of high quality RNA, that is, the tissue must have been frozen immediately after collection and must not have been fixed by formalin. Taken together, our samples are—to our knowledge—unique.

Importantly, clone TCR‐IP2 was not only expanded in the CSF but also in the hippocampus of patient IP2, and about half of the hippocampus‐resident CD8+ T cells expressed the activation marker perforin. By contrast, despite a considerable number of infiltrating T cells, clone TCR‐IP2 could not be detected in the OIC except a single read in NGS. This preference for the hippocampus together with its monoclonal expansion, parenchymal localization, and perforin expression underlines that TCR‐IP2 may indeed play an important role in the pathogenesis of GABA_A_‐R encephalitis. Moreover, clonally expanded TCR‐IP2 cells were present in the CSF and hippocampus despite intense medication with immunosuppressants^6^ indicating sustained, probably antigen‐driven activation. Taken together, clonally expanded TCR‐IP2 cells are therefore likely not a mere response to seizures and epilepsy.^20^

In autoimmune encephalitis, much attention has been paid to analyses of antibodies, whereas T cell responses have rarely been studied.^21^, ^22^, ^23^, ^24^ We here found parallel intrathecal and parenchymal expansions of a likely pathogenic CD8+ T cell clone supporting the assumption that T cells have so far been unjustifiably disregarded. The link between the dominant role of autoantibodies and concomitant invasion of putatively pathogenic T cell clones requires further investigation in order to reveal the synergy between both arms of the adaptive immune system.

## Conflict of Interest

The authors declare no competing financial interests.

## Author Contributions

AB performed IHC, LCM, PCR, and TCR analysis experiments, E.B. performed PCR and NGS experiments, CA, JF, and BC provided samples and clinical data and contributed to data analysis, NM and RH contributed to data analysis and writing of the manuscript, KD, and EB initiated and designed the research, analyzed the data, and wrote the paper. All authors edited, reviewed, and approved the manuscript.

## Supporting information


**Data S1** **.** Immunohistochemistry and isolation of single T cells from brain parenchyma.
**Table S1.** Clone‐specific PCR primers for amplification of TCR chains on clone TCR‐IP2 by nested PCR.
**Table S2.** TCR chains identified in single CD4^+^ T cells from CSF of IP2.
**Table S3.** TCR chains identified in single CD8^+^ T cells from CSF of IP2.
