# Supplementary material for: An expanded parenchymal CD8+ T cell clone in GABAA receptor encephalitis
Source: Ann Clin Transl Neurol. 2020 Jan 14;7(2):239–44. doi: 10.1002/acn3.50974 (PMC7034500; doi:10.1002/acn3.50974)
Supplement: Supplementary file 1 — Data S1 . Immunohistochemistry and isolation of single T cells from brain parenchyma. Table S1. Clone‐specific PCR primers for amplification of TCR chains on clone TCR‐IP2 by nested PCR. Table S2. TCR chains identified in single CD4+ T cells from CSF of IP2. Table S3. TCR chains identified in single CD8+ T cells from CSF of IP2. [file ACN3-7-239-s001.docx]

Supplementary Material

**Short Communication**

**An expanded parenchymal CD8^+^ T cell clone in GABA-A receptor encephalitis**

Aline Bracher, Carmen Alcalá, Jaime Ferrer, Nico Melzer, Reinhard Hohlfeld,

Bonaventura Casanova, Eduardo Beltrán, and Klaus Dornmair

This supplementary material is composed of:

- Supplementary Methods

- Supplementary Table 1

- Supplementary Table 2

- Supplementary Table 3

**Supplementary Methods:**

Immunohistochemistry and isolation of single T cells from brain parenchyma

For IHC, FFPE fixed brain tissue from patient IP2 was deparaffinized by xylene and stepwise rehydration. Heat-induced epitope retrieval was performed at 95 °C for 30 min in Tris-EDTA buffer. Tissue was permeabilized by 0.05 % Triton, and blocked for 1 h with 10 % fetal bovine serum and 1 % bovine serum albumin. Samples were double stained for CD3 and CD4 or CD8 with polyclonal rabbit anti-human CD3 (Dako, Hamburg, Germany, 1:50), mouse anti-human CD8 (clone C8/144B, Dako, 1:50) or mouse anti-human CD4 (clone 4B12, Dako, 1:50), and the secondary antibodies goat anti-rabbit IgG-AF594 (ThermoFisher) and goat anti-mouse IgG1κ-AF488 (ThermoFisher). After washing, 4′,6-diamidin-2-phenylindole (DAPI, 1:1000) was added.

For IHC on frozen tissue sections, tissue slices were fixed with acetone, washed, blocked with BSA, and stained first with an anti-human perforin antibody (clone B-D48, Abcam, Darmstadt, Germany, 1:100), followed by the secondary goat anti-mouse IgG1-AF488 antibody (ThermoFisher, 1:1000), washed, and then stained with anti-human CD8α-Cy3 (clone LT8, AbD-Serotec, Puchheim, Germany, 1:50) and finally with DAPI (1:1000).

For laser capture microdissection, 10 µm sections of hippocampus or OIC were mounted on 1.0 PET foil slides, fixed with acetone, washed, and stained with 100 µl anti-CD4-AF488 (clone RPA-T4, BD Bioscience, Heidelberg, Germany, 1:100) for 5 minutes. After washing, cells were stained with 100 µl anti-CD8-Cy3 (clone LT8, AbD-Serotec, 1:50) for 3 minutes. After washing, samples were covered with 250 µl 2-propanol and screened for CD4+ and CD8+ T cells using an Axiovert 200 M microscope with Robo Software V3.2.0.11 (Zeiss-PALM). After drying, cells were individually collected and stored on dry ice.

**Supplementary Table 1: Clone-specific PCR primers for amplification of TCR chains on clone TCR-IP2 by nested PCR.**

**1^st^ PCR:**

Vα-8,21-for-out and Cα-out ref. [12]

VP7 and Cβ-mid4 ref. [13]

**2^nd^ PCR:**

Vα8/1-for-in and Cα-rev-in ref. [12]

VP7 and Cβ-in ref. [13]

**3rd PCR:**

N05-13-Vα-8.1-spec-for 5‘ AGACATTCGTTCAAATGTGGG

N05-13-Vα-8.1-spec-rev 5‘ ATAGAACTGGTTACCAGTGCC

N05-13-Vβ-8.1-spec-for 5‘ AGATCCAGCCCTCAGAACC

N05-13-Vβ-8.1-spec-rev 5‘ GCCAAAATACTGCGTCTCTCC

Supplementary Table 1

**Supplementary Table 2: TCR chains identified in single CD4^+^ T cells from CSF of IP2.** TCR β-chains and, if detected, corresponding α-chains are listed. The clone numbers (column 1) and chain designation (column 2), TRV (column 3) and TRJ (column 4) regions, the amino acid sequences of their CDR3- regions (column 5), and the frequencies (column 6) are given. In total, β-chains could be identified from 15 single cells. In 11 of these cells, matching α-chains could also be identified.

1 α 24 45 CAF-MG-GGGADGLTFGKG 1

β 4-2 1-1 CASS-LKTGGYL-NTEAFFGQG 1

2 α 21 58 CAVR-VV-ETSGSRLTFGEG 1

α 17 12 CATD-Q-MDSSYKLIFGSG 1

β 19 1-1 CASS-WPGQG-NTEAFFGQG 1

3 α 12-1 44 CAVV-SP-TGTASKLTFGTG 1

β 6-1 1-1 CASS-SLQGSPRG-NTEAFFGQG 1

4 α 24 41 CA-FPGR-SGYALNFGKG 1

β 6-5 1-2 CAS-RSGD-NYGYTFGSG 1

5 α 8-2 53 CAV-SAR-GGSNYKLTFGKG 1

β 7-8 2-1 CASS-IKNP-YNEQFFGPG 1

6 α 12-2 54 CAM-RL-QGAQKLVFGQG 1

β 3-1 1-1 CASS-QGSWARM-NTEAFFGQG 1

7 α 13-2 4 CAEN-P-GGYNKLILEQGPG 1

β 28 1-5 CASS-IEG-NQPQHFGDG 1

8 α 38-2 27 CAYRS-GP-AGKSTFGDG 1

β 16 1-4 CASSQ-PLAGS-TEKLFFGSG 1

9 α 26-1 20 CIV-SV-SNDYKLSFGAG 1

β 6-6 1-2 CASS-YTEAS-YGYTFGSG 1

10 α 12-3 21 YLC-VMRG-NFNKFYFGSG 1

β 12-3 2-3 CASS-WGV-TDTQYFGPG 1

11 α 12-2 49 CAV-TP-NTGNQFYFGTG 1

β 6-5 1-2 CAS-TDSTGTGA-NYGYTFG 1

12 β 5-1 1-1 CASS-FGAR-TEAFFGQG 1

13 β 12-3 2-5 CAS-TLRGT-ETQYFGPG 1

14 β 12-3 2-1 CASS-FRLAGY-YNEQFFGPG 1

15 β 28 1-2 CAS-TLRDS-YGYTFGSG 1

Supplementary Table 2

**Supplementary Table 3: TCR chains identified in single CD8^+^ T cells from CSF of IP2.** In total, β-chains could be identified from 13 single cells. In 11 of these cells, a matching α-chain could also be identified. See legend to Suppl. Table 1 for details.

1 to 6 α 13-1 49 CAAS-WG-TGNQFYFGTG **6**

β 12-3 2-3 CASS-AGG-DTQYFGPG **6**

7 α 41 48 CAV-IGRGP-NFNEKLTFGTG 1

β 12-3 1-6 CASS-FLDK-NSPLHFGNG 1

8 α 10 5 CAVVS-AE-DTGRRALTFGSG 1

β 5-1 1-5 CASS-QGPQ-SNQPQHFGDG 1

9 α 12-3 20 CAMS-VD-DYKLSFGAG 1

β 7-9 1-4 CAS-TVA-TNEKLFFGSG 1

10 α 8-3 30 CAVGA-P-NRDDKIIFGKG 1

β 12-4 2-6 CASS-LSSD-GANVLTFGAG 1

11 α 12-2 4 CAVN-SQ-GGYNKLIFGAG 1

α 35 53 CA-AS-SGGSNYKLTFGKG 1

β 3-1 1-6 CASSQ-DTGR-SPLHFGNG 1

20 β 7-2 2-5 CASS-PGQG-QETQYFGP 1

21 β 7-8 1-5 CASSL-AEGY-SNQPQHFGDG 1

Supplementary Table 3
